# Transcriptomic Responses of *Cordyceps militaris* to Salt Treatment During Cordycepins Production

**DOI:** 10.3389/fnut.2021.793795

**Published:** 2021-12-23

**Authors:** Gongbo Lv, Yue Zhu, Xiaojie Cheng, Yan Cao, Bin Zeng, Xinping Liu, Bin He

**Affiliations:** ^1^Jiangxi Key Laboratory of Bioprocess Engineering and Co-innovation Center for in-vitro Diagnostic Reagents and Devices of Jiangxi Province, College of Life Sciences, Jiangxi Science and Technology Normal University, Nanchang, China; ^2^College of Life Sciences, Sichuan Normal University, Chengdu, China; ^3^Information Institute of Sichuan Academy of Agricultural Sciences, Chengdu, China; ^4^College of Pharmacy, Shenzhen Technology University, Shenzhen, China

**Keywords:** cordycepin, *Cordyceps militaris*, salt treatment, molecular mechanism, transcriptome

## Abstract

Cordycepin is a major bioactive compound found in *Cordyceps militaris* (*C. militaris*) that exhibits a broad spectrum of biological activities. Hence, it is potentially a bioactive ingredient of pharmaceutical and cosmetic products. However, overexploitation and low productivity of natural *C. militaris* is a barrier to commercialization, which leads to insufficient supply to meet its existing market demands. In this study, a preliminary study of distinct concentrations of salt treatments toward *C. militaris* was conducted. Although the growth of *C. militaris* was inhibited by different salt treatments, the cordycepin production increased significantly accompanied by the increment of salt concentration. Among them, the content of cordycepin in the 7% salt-treated group was five-fold higher than that of the control group. Further transcriptome analysis of samples with four salt concentrations, coupled with Gene Ontology (GO) analysis and Kyoto Encyclopedia of Genes and Genomes (KEGG) pathway enrichment, several differentially expressed genes (DEGs) were found. Finally, dynamic changes of the expression patterns of four genes involved in the cordycepin biosynthesis pathway were observed by the quantitative real-time PCR. Taken together, our study provides a global transcriptome characterization of the salt treatment adaptation process in *C. militaris* and facilitates the construction of industrial strains with a high cordycepin production and salt tolerance.

## Introduction

*Cordyceps militaris* (*C. militaris*), or known as North *Cordyceps sinensis*, is an entomogenous fungus belonging to the *Ascomycota, Hypocreales*, and *Ergotacea*e, as spores are produced internally inside a sac, called ascus (1). It is a well-known edible and medicinal fungus and one of the most important traditional Chinese medicines. Contrary to *Cordyceps sinensis* (DongChongXiaCao) in Chinese herbs, which is unique to China, *C. militaris* is a worldwide species and is also distributed in almost all provinces and regions in China. Its soporophore can parasitize larvae or grow on the pupae of lepidopteran insects. These insects are located on the scales half-buried on the forest floor or under the layer of deciduous branches from spring to autumn (2). *C. militaris* has been extensively used as a folk medicine in areas of East Asia for the revitalization of various systems of the body from ancient times, and currently it is also widely used in Western countries (3). Since *C. militaris* produces a variety of bioactive compounds with functional properties, it has long been utilized as a dietary supplement (tonic food) or herbal medicine for the pharmaceutical and cosmetics industries for many years (4, 5). Various bioactive metabolites isolated from *C. militaris*, such as cordycepin (3′-deoxyadenosine) (6), cordycepic acid, carotenoid, ergosterol, and cordyceps polysaccharide (7), have been explored extensively for extending its applications; particularly, several of them are extracted and made into tablets and capsules (8). In addition, fermentation and downstream processes or other bioprocess developments have permitted a prospect in the production of these specific bioactive metabolites as functional ingredients for diversified applications (9).

Among the aforementioned bioactive metabolites, the cordycepin (3′-deoxyadenosine) has attracted the most attention in *C. militaris*, and its potential therapeutic effect has been mostly mentioned and investigated for clinical trials against cancer (10). As the main bioactive ingredient of *C. militaris*, cordycepin is used as a transcription inhibitor for its lack of hydroxyl moiety at the C3 position. When cordycepin is integrated into the RNA chain, it will lead to the termination of transcription (11). Moreover, it has been reported that cordycepin can inhibit cell proliferation and induce cell apoptosis *via* binding signaling molecules, which resulted in anti-inflammatory action (12–14). Cordycepin and adenosine have a similar structure, except for the lack of a 3′ hydroxyl group on cordycepin. Even with such a tiny difference, cordycepin and adenosine exhibit completely distinct biological activity, and cordycepin is reported to interfere with many molecular and cellular processes within cells. Furthermore, extensive research results showed that cordycepin has many other diversified biological activities, such as a very potent anti-cancer, anti-ischemic, and anti-oxidant protective effect. Exactly, cordycepin binds the A3 adenosine receptor, which results in activating G protein (inhibitory regulative guanine nucleotide-binding protein). Afterward, the cAMP formation was inhibited, then the serine/threonine kinase glycogen synthase kinase (GSK)-3β/β-catenin signaling pathway and subsequently cell division were indirectly inactivated and suppressed, respectively (15). Additionally, it has been also documented that cordycepin effects on inhibiting platelet aggregation, inducing steroid formation, and with broad-spectrum antibiotic activity by inhibiting NAD^+^-dependent DNA ligase (13, 16, 17).

With over seven-decade-long historical investigations of cordycepin, it was first isolated from *C. militaris* in 1950 (6), and the chemical structure of cordycepin was confirmed to be 3′-deoxyadenosine *via* mass spectrometry data, infrared spectroscopy, and nuclear magnetic resonance spectroscopy data, combined with the characteristics of the ultraviolet absorption spectrum (18, 19). Meanwhile, the cordycepin biosynthetic pathway has been exploited for over half-centuries. Though several hypotheses concerning cordycepin biosynthetic pathways were proposed, clear experimental evidence to clarify “how cordycepin is produced” was lacking. With the completion of the whole genome sequencing of *C. militaris*, Xia et al. first identified the gene cluster responsible for the cordycepin biosynthesis, and the cordycepin biosynthesis pathway was further fulfilled in 2017 (20, 21). There were four genes (*Cns1–Cns4*) physically linked as the cordycepin biosynthetic gene cluster and encoded proteins with distinct conserved domains, which differently mediated cordycepin metabolism. Among them, *Cns1* (CCM_04436) contains the oxidoreductase/dehydrogenase domain, and *Cns2* (CCM_04437) possesses the HDc family of metal-dependent phosphohydrolase domain. There were two functional domains contained in *Cns3* (CCM_04438): an N-terminal nucleoside/nucleotide kinase (NK) and a C-terminal HisG family of ATP phosphoribosyltransferases. Lastly, *Cns4* (CCM_04439) was identified as a member of the putative pleiotropic drug resistance (PDR) family of ATP-binding cassette (ABC) transporters (20). According to the finding of Xia et al., cordycepin biosynthesis begins at adenosine, and the hydroxyl phosphorylation is catalyzed by *Cns3* to produce adenosine-3′-monophosphate (3′AMP). Subsequently, 3′AMP was dephosphorylated to 2′-carbonyl-3′-deoxyadenosine (2′-C-3′-dA) by phosphohydrolase activity of *Cns2* (21). Finally, the 2′-C-3′-dA was converted to cordycepin *via* oxidoreduction reactions mediated by *Cns1*. Simultaneously, it was noteworthy that pentostatin (PTN, 2′-deoxycoformycin), an irreversible inhibitor of adenosine deaminase, was yielded in coupling with cordycepin by phosphoribosyltransferase domain of *Cns3* (22). Consequently, this process inhibited the deamination of cordycepin to 3′-deoxyinosine to maintain the stability of cellular cordycepin through the bacterium protector-protégé strategy (23). Furthermore, when cordycepin accumulation reaches a high (toxic) intracellular level, such PTN will be pumped out of the cell by the *Cns4* transporter for neutralizing cordycepin to nontoxic 3′-deoxyinosine (24).

To meet the existing and increasing market demand of cordycepin production, strategies for the enhancement of this medical component production in *Cordyceps* spp. are warranted. Plentiful literature has been reported on diversified approaches which focused on the improvement of cordycepin content, such as *C. militaris* strain mutagenesis, optimization of medium composition, culture conditions, and extraction methods. Among them, optimization of medium composition is usually the first choice to increase the content of cordycepin. Growth supplements and additives, such as sugar, amino acids (L-alanine, glycine, casein hydrolysate, and glutamine), vitamins, inorganic salts (ferrous sulfate and sodium selenite), nucleoside analog (hypoxanthine and adenosine), porcine liver extracts, or vegetable oils (peanut and cottonseed oil), were proved to be successful (8, 25–30). Moreover, ways of genetic and metabolic engineering are noteworthy, and two main methods are mentioned as follows, the overexpression and disruption of the gene on the cordycepin biosynthetic cluster. It has been reported that the production of cordycepin was increased by 25% by deletion of *Cns3* when compared with the control (21). Meanwhile, overexpression of the single *Cns1, Cns2*, and *Cns3* along with the *Cns1*/*Cns2* fusion gene was also performed *via* individually transformed the control strain of *C. militaris*. It was observed that in contrast to the wild-type strain, the yield of cordycepin in the transformant was engineered with the *Cns1*/*Cns2* fusion gene that was increased 2.7-fold, whereas no obvious differences were found between the control and transformants engineered with single genes. Moreover, previous reports showed that cordycepin production could be increased by ultraviolet, ^60^Co γ-ray, and blue light LED irradiation. For instance, the cordycepin titer of *Paecilomyces hepiali* ZJB18001strain was increased 2.3-fold *via* ^60^Co γ-ray and ultraviolet irradiation compared with that from the wild strain (27, 29, 31). Additionally, nutrient stress (distinct carbon, nitrogen, phosphorous sources, and ratios) and environmental stress (lighting time, temperature, pH, and shaking speed) were also effective strategies. It was reported that glucose and casein hydrolysate were the most beneficial carbon and nitrogen sources in cordycepin production (2.3-fold improvement relative to that of the control), and especially, production was greatly affected by casein hydrolysate (29).

Salt stress has negative effects on the growth and survival of organisms, such as leading to water loss and cell shrinking, and several metabolic changes are observed (32, 33). However, to the best of our knowledge, a detailed mechanism of the response of *C. militaris* to salt treatment and its effect on the enrichment of cordycepin remains to be elucidated. The high-salt fermenting environment prolongs the process of substrate catabolism and biosynthesis for the decrease in cell growth and enzyme performance. Thus, the construction of industrial strains with a high cordycepin production and salt tolerance for industrial production is highly desirable. To better comprehend the molecular mechanism of salt-action on cordycepin production, a transcriptome analysis was carried out in the present study. Our study demonstrated the effect of different salt concentrations on cordycepin contents in the CM01 strain, followed by a transcriptome analysis based on RNA sequencing of each salt-treated *C. militaris*, coupled with gene oncology (GO) analysis and Kyoto Encyclopedia of Genes and Genomes (KEGG) pathway enrichment of differentially expressed genes (DEGs). Generally, there is an array of genes that regulates the synthesis of bioactive metabolites, and these genes were upregulated or downregulated during the biosynthesis of cordycepin. Therefore, mRNA expression analysis of several genes involved in the cordycepin biosynthesis pathway was also performed to determine the effect of different salt treatments on potential target genes whose modulation may increase cordycepin production to meet the industrial needs.

## Materials and Methods

### Fungal Strain and Cultivation

In this study, the *C. militaris* strains involved in the experiments was the commonly used strain in our laboratory, CM01strain. This strain is an isolate of *C. militaris* with a mating type of *M4T1-1*. A basal medium, containing (g/L) glucose 20, peptone 5, KH_2_PO_4_ 1, MgSO_4_·7H_2_O 0.5, Vitamin B_1_ 0.05, and Vitamin B_2_ 0.05, was used. In total, 200 g potato was milled and boiled with water and filtrated with gauze. The filtrates were mixed with the basal medium, and then water was added to the final volume of 1,000 ml to prepare the liquid medium. After that, the pH of the liquid medium was adjusted to 7.2. The prepared liquid medium was divided into 250 ml flasks (100 ml per flask), coupled with 2 g agar, and one of them was not added agar for strain activating. In total, 0, 3, 5, and 7 g NaCl per 100 ml were added to the medium as treatments, which were equivalent to control, slight stress, moderate stress, and severe salt treatment, respectively. And then all of the media were sterilized in moist heat sterilization autoclave for 20 min at 121°C. When the culture medium was cooled, *C. militaris* strain CM01 was incubated in the prepared liquid medium for activating strain under aseptic conditions. The vibration culture with a speed of 120–140 rpm/min was employed to expand the culture when the white mycelium grows from the seed block (generally 24 h). The activated strain was followed by inoculation on distinct salt-treated potato dextrose agar (PDA) plates and cultured in darkness at a temperature of 22°C. The culture was incubated for 5–7 days. After the media were covered by mycelia, these plates were transferred to the light culture at a temperature of 22°C and lighting intensity of 600 lx for 15 h each day (about 5 days). It was followed by scraping and then drying the *C. militaris* mycelia overnight at 60°C for the biomass determination. All experiments were performed in triplicate to ensure reproducibility.

### Extraction of Cordycepin From *C. militaris*

After 5 days of light culture, *C. militaris* mycelium was harvested, and then cordycepin content was determined for the next analysis. Specifically, the mycelia was lyophilized, vacuum freeze-dried to a constant weight, and ground to powder. Two grams of dry powder were weighted from each sample to perform High Performance Liquid Chromatography (HPLC) detection. HPLC assay was conducted on Waters Alliance e2695 HPLC (Milford, MA, USA) using a UV detector set at 260 nm equipped with a ZorbaxSB-C18 column (4.6 × 250 mm, 5 μm). The analysis conditions were as follows: mobile phase, 85% ultra-pure water/methanol (85:15, v/v), and the elution rate was 1.5 ml min^−1^; injection volume, 20 μl. A standard cordycepin curve was generated using 0.02–0.25 μg/ml cordycepin standard (Sigma-Aldrich, Burlington, MA, USA). The cordycepin yield was calculated using the detected peak area according to the standard curve. The cordycepin concentration of mycelia presented in our study was calculated by normalizing in the equal biomass.

### Extraction of Total RNA, Library Establishment, and Transcriptome Sequencing

Total RNA extraction was performed with a fungal RNA kit (Omega Bio-tek, Norcross, GA, USA) by following the operating instructions, coupled with DNA digestion. NanoDrop ND-1000 spectrophotometer (Thermo Scientific, Wilmington, DE, USA) and Bioanalyzer 2100 (Agilent Technologies, Palo Alto, CA, USA) were employed to analyze RNA concentration and integrity. Thereafter, mRNA was enriched by Oligo (dT) beads. The mRNAs were fragmented and used as templates to synthesize cDNA. The cDNA fragments were purified using QiaQuick PCR extraction kit, end-repaired, single-nucleotide adenine addition, and ligated to Illumina sequencing adapters to create the cDNA library. The size of the ligation products was selected by agarose gel electrophoresis, amplified, and sequenced using Illumina HiSeqTM2500 (Biomarker Biotechnology Co., Beijing, China) (34). RNA-Seq data of *C. militaris* under salt treatment were deposited in the NCBI/SRA database (https://www.ncbi.nlm.nih.gov/sra), under the Bioproject accession number PRJNA770191; BioSample: SAMN22211876-SAMN22211879.

### Transcriptome Analysis

The transcriptome datasets are raw reads containing adapters or low-quality bases. Therefore, reads will be further filtered by our previous criterion to get clean reads and thus removed (35). Moreover, Bowtie2 software (http://bowtie-bio.sourceforge.net/bowtie2) was performed to remove reads that mapped to ribosome RNA (rRNA) database to get the final clean reads, which were further employed for assembly and transcriptome analysis (36). For gene expression analysis, the obtained clean-read data sets of the four different cultures were mapped to the reference genome *C. militaris* CM01 (NCBI accession number. AEVU00000000) using Tophats2 (v2.0.3.12) (37). RSEM software was used to quantify gene abundances, and the quantification of gene expression level was normalized using the Fragments Per Kilobase of transcript per Million mapped reads (FPKM) method (38, 39).

### DEGs Annotation

Differentially expressed genes across samples were identified using edgeR on the R package (version 3.4.2) according to the RSEM results (40). The absolute value of fold change ≥2 and false discovery rate (FDR) within 0.05 were set as the threshold for the detection of significant DEGs among four treatments (CK vs. NaCl-3, -5, and -7) (34). Then the identified DEGs were carried out into hierarchical clustering, with the KEGG pathway enrichment analysis. To further analyze the DEG annotations, GO functional classification and Kyoto Encyclopedia of Genes and Genomes (KEGG) pathway enrichment analysis were carried out based on the GO database (41) and KEGG database (42).

### Quantitative Real-Time PCR Validations of DEGs

To validate the transcriptional level results from RNA-Seq data analysis, four genes (encode cordycepin synthetase) *Cns1, Cns2, Cns3*, and *Cns4* are involved in the cordycepin biosynthesis in *C. militaris* were selected for real-time RT-PCR validation. The qRT-PCR template cDNA was synthesized from 0.5 μg of total RNA by Fungal RNA Kit. Reverse transcription of each RNA sample was performed to get the first-strand cDNA using the PrimeScript RT reagent Kit with gDNA Eraser (Takara, Dalian, China). Each qRT-PCR reaction system had a total volume of 10 μl, containing cDNA, relevant primers, and the SYBR Green Real-time PCR Master Mix (Takara). Real-time RT-PCR was performed using Real-Time PCR System (Bio-Rad, Hercules, CA, USA). GAPDH was served as the internal control (reference gene) for normalization of the target gene expression and to correct for variation between samples. The thermal cycle for RT-PCR was as follows: 95°C for 2 min, followed by 40 cycles of 95°C for 10 sec, 60°C for 15 sec, and 72°C for 20 sec. Melting curve analyses of the amplification products were performed at the end of each PCR reaction to ensure that only specific products were amplified. Primers used for the candidate genes are designed according to Illumina sequencing data by using Primer Premier 5 and listed in Supplementary Table S1. The comparative 2^−ΔΔCT^ method was employed to calculate relative expression levels between the target genes.

### Data Analysis

Three independent experiments were performed, and all the data in this study are presented as mean ± SE of three replicates. Data from the same period were evaluated by one-way nested ANOVA, followed by the least significant difference test (LSD) for mean comparison. One tail student *t*-test was conducted between the control and salt-treated groups to calculate the *p*-values. All statistical analysis was performed with SAS 9.20 software (SAS Institute Inc., Cary, NC, USA) at the *p* < 0.05.

## Results

### Growth Characteristics and Cordycepin Production From *C. militaris* After Salt Treatments

**Figure 1:**
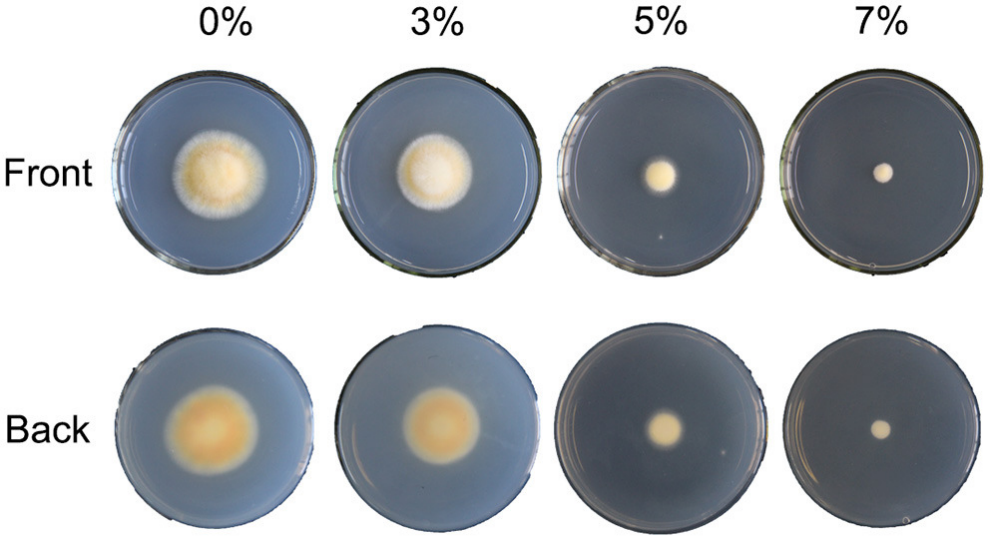
The phenotype of *C. militaris* is affected by different salt treatments. 0, 3, 5, and 7% are equivalent to the control, slight, moderate, and severe salt treatment, respectively.

To explore the effects of salt treatment on cell growth and cordycepin production of *C. militaris*, a preliminary study of distinct concentrations of salt treatments was conducted. After strain activation, incubation, and dark and light culture, the phenotypes of *C. militaris* mycelioid colonies of the salt-treated group were significantly different from the control (Figure 1). As the result showed, the growth of *C. militaris* was significantly restricted by salt treatment and the colony size descends remarkably as the salt concentration increases, especially under the 5 and 7% salt treatments. When the salt concentration is beyond 7%, the mycelia of *C. militaris* cannot even grow at all. It was followed by determining the biomass of the *C. militaris* mycelia. According to the obtained results, the biomass was decreased with the increase of salt concentration (Supplementary Figure S1). Analogous to colony diameter, the lowest biomass of the *C. militaris* mycelia was found in the 7% salt-treated group.

**Figure 2:**
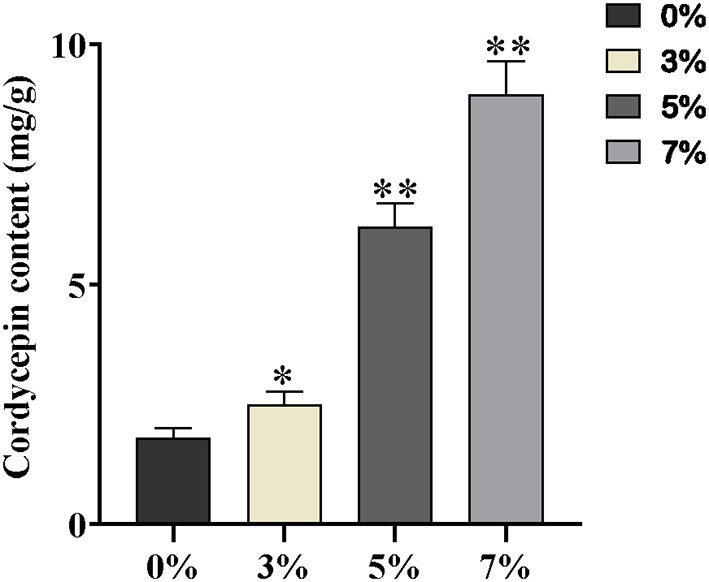
The contents of cordycepin with mg/g dry weight 0, 3, 5, and 7% are equivalent to the control, slight, moderate, and severe salt treatment, respectively. Asterisks indicate statistically significant differences between groups (Student's *t*-test): * and ** indicating significance level was accepted at *P* ≤ 0.05 and *P* < 0.01, respectively, as compared to control.

High Performance Liquid Chromatography detection assay was performed to determine the production of cordycepin of each salt-treated group. Although the growth of *C. militaris* was inhibited by distinct salt treatments, the cordycepin production of per unit volume of mycelium has increased notably, accompanied by the improvement of salt concentration (Supplementary Figure S2). Noteworthily, the content of cordycepin treated with 7% salt was five-fold higher than that of the control group, and the remaining cordycepin content with mg/g dry weight is presented in Figure 2.

### Transcriptome Overview

To investigate the molecular mechanism of salt treatment that increases the per-volume content of cordycepin in *C. militaris*, a transcriptome analysis based on RNA sequencing of each salt-treated *C. militaris* was performed. Four cDNA samples from *C. militaris* mycelia were processed by the Illumina HiSeq platform, and the transcriptome data obtained by sequencing results were listed in Table 1. This resulted in the generation of 40.81, 40.50, 45.22, and 55.58 million clean reads per library, respectively. The GC content for all treatments was ~57%, and the % ≥Q30 (99.9% accuracy of bases) was >94% for all samples, indicating a good quality of the sequencing data which can be used to do the following analysis. After mapping the sequenced reads to the reference genome, more than 92.5% of the reads in all four samples were mapped. Additionally, a total of 134,436, 155,697, 159,689, and 162,615 single nucleotide polymorphism (SNP) numbers were generated, and most of them were synonymous mutations according to the subsequent annotation.

**Table 1:** Summary of the sequencing data of *C. militaris* under different salt concentrations.

**Samples**	**Control**	**NaCl-3**	**NaCl-5**	**NaCl-7**
Clean reads	40817724	45502268	45225634	55588246
GC Content	57.51%	57.26%	57.30%	57.57%
Q30	94.47%	95.00%	94.28%	94.74%
Mapping rates	92.52%	92.71%	93.01%	93.79%
SNP number	134436	155697	159689	162615
Genic SNP	102703	112702	115509	116064
Intergenic SNP	31733	42995	44180	46551

### DEGs Analysis

**Figure 3:**
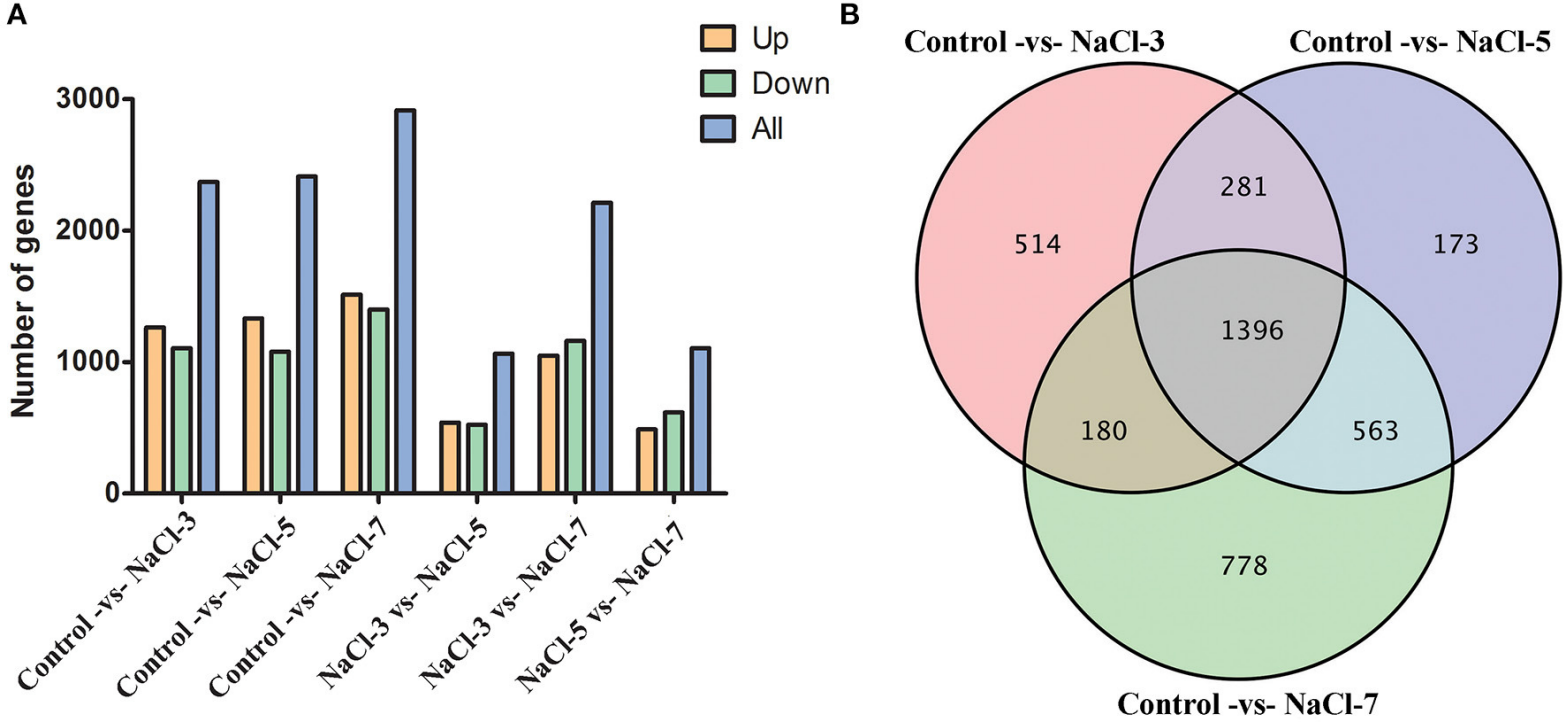
Distribution of differentially expressed genes (DEGs) in the six samples. **(A)** DEGs' distribution between control and three different salt treatments; **(B)** Venn diagram of the commonly expressed genes between samples. NaCl-3, 5, and 7 represent 3, 5, and 7% salt concentrations, respectively.

To identify genes with altered expression levels with salt treatment, the overall transcription levels of genes were quantified by Revenue Passenger Kilometers (RPKM) metrics. According to global transcriptional changes from normalizing the DEG data (Supplementary Table S2), a total of 3,885 genes showed altered expression levels in the three salinity treatment groups, as compared to the control. There were 2,917 DEGs in the Control vs. NaCl-7 group, most of all groups, 1,501 upregulated genes (51%) and 1,416 downregulated genes (49%); followed by 2,413 and 2,371 DEGs in the Control vs. NaCl-5 and Control vs. NaCl-3 groups, separately. In addition, there were no significant differences between NaCl-3 vs. NaCl-5 and NaCl-5 vs. NaCl-7 groups (Figure 3A). Venn diagram analysis of DEGs between the control and salt treatment treatments revealed that 1,396 DEGs were commonly shared among the distinct salt treatments, the other commonly shared DEGs between every two groups were 180, 281, and 563, respectively (Figure 3B). Moreover, a heatmap was drawn to present the hierarchical clustering of the DEGs among samples (Supplementary Figure S3). As shown in the heatmap, the expression profiles between the control and three salt-treated groups were different, illustrating that the effect of different salt concentrations on *C. militaris* is distinct GO functional classification of DEGs.

**Figure 4:**
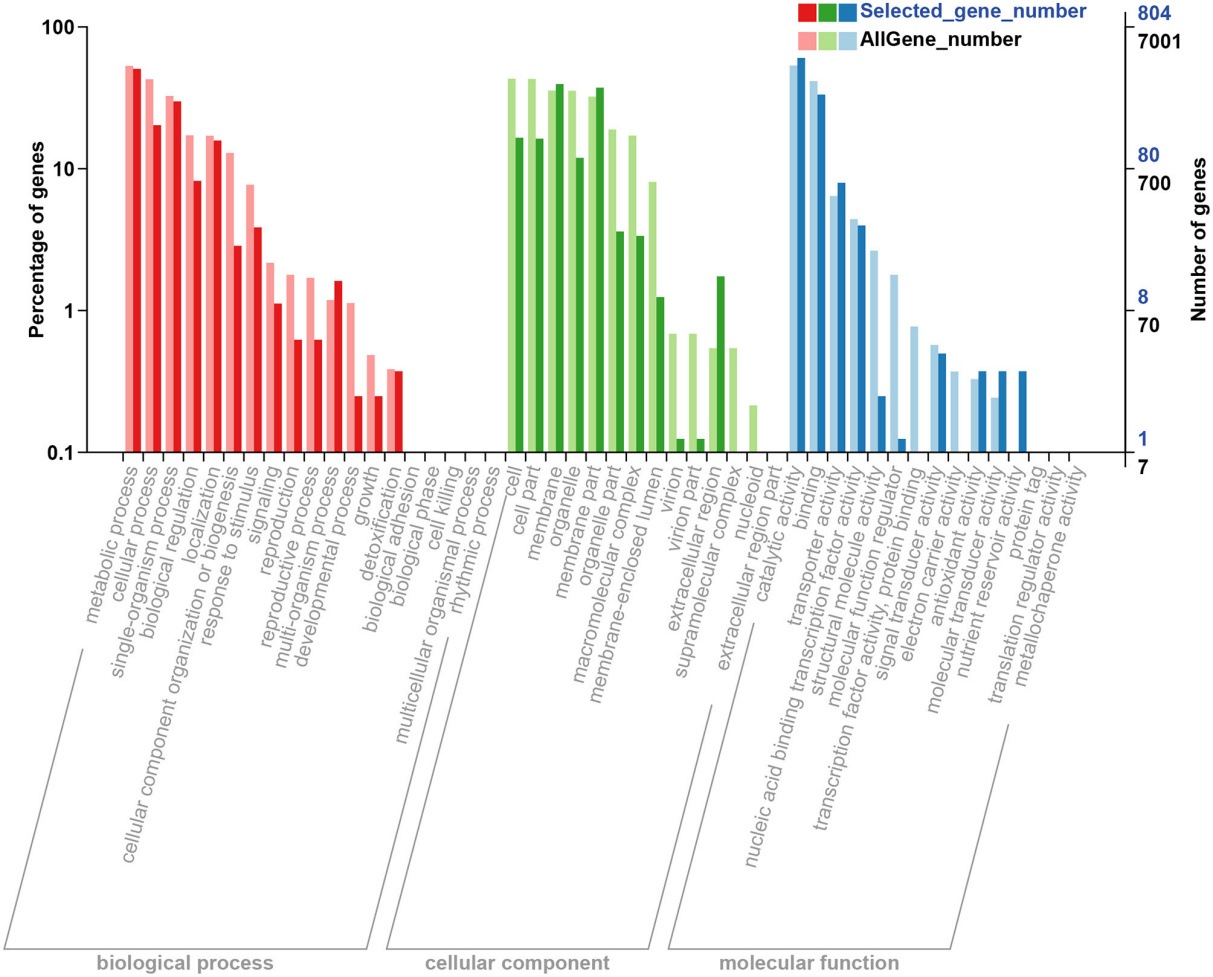
GO classification and enrichment of the DEGs based on the GO database [the *x*-axis represents the GO term and enrichment, and the *y*-axis represents the percentage of genes (left) and the number of DEGs (right)]. Selected_gene_number represents the number of DEGs. GO, gene oncology; DEGs, differentially expressed genes.

To analyze the function of DEGs and gene annotation on the control and salt treatment strains, GO classification and functional enrichment were performed. Generally, GO annotation consists of three types: biological process, cellular component, and molecular function. Based on GO annotation, a total of 1,096, 1,060, and 866 DEGs were assigned to the ontologies “biological process”, “cellular component”, and “molecular function”, respectively; for the biological process classification, most of DEGs focused on “metabolic process”, “cellular process”, and “single-organism process” item, etc. In the part of cellular component, “cell”, “membrane”, and “membrane part” were the most highly represented categories; while for the molecular function classification, “catalytic activity” was the most enriched GO term, followed by “binding”, and “transporter activity”, and the detailed distribution of GO terms are illustrated in Figure 4.

### KEGG Pathway Enrichment of DEGs

**Figure 5:**
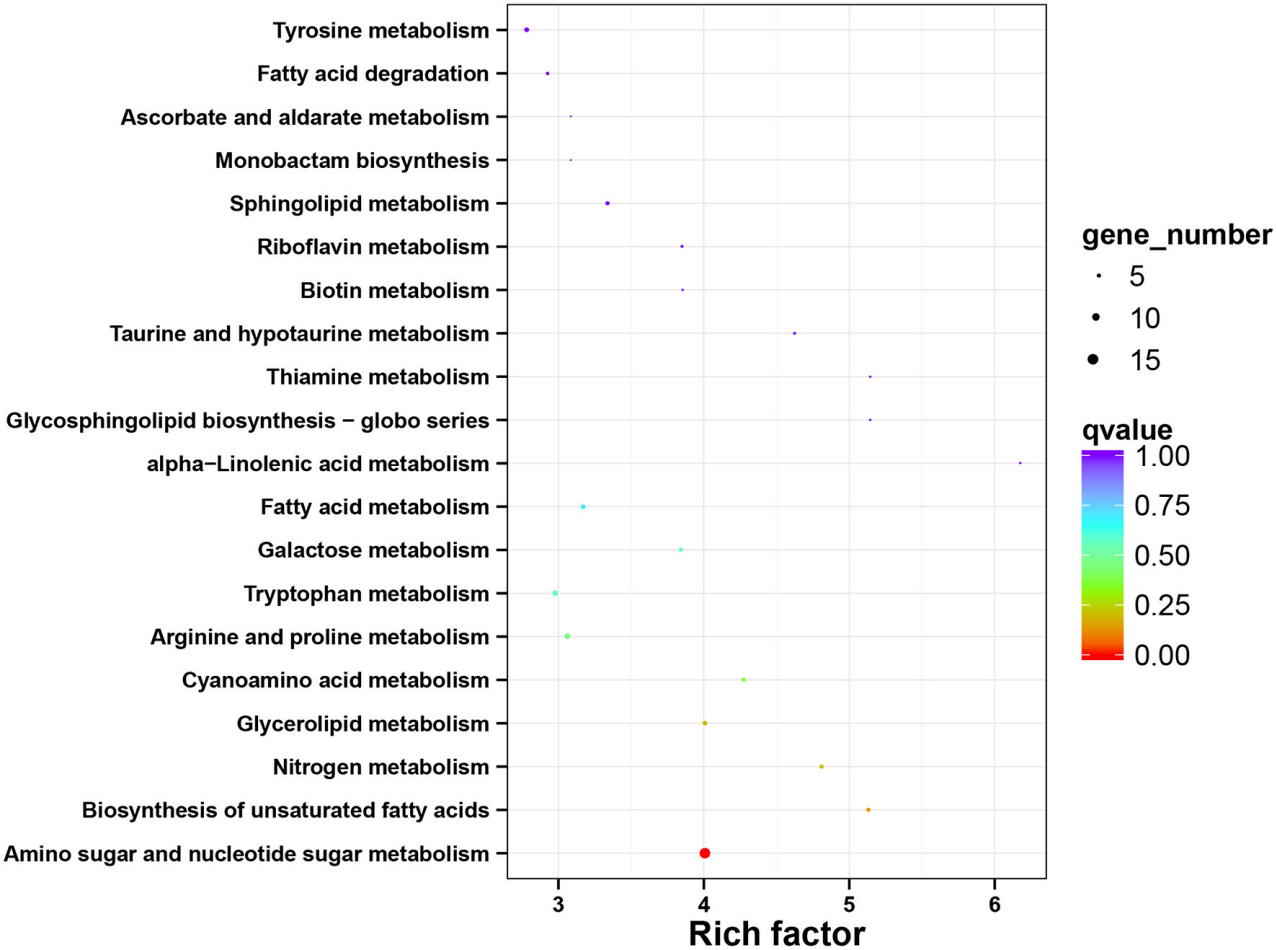
The functional enrichment analysis of DEGs using KEGG annotation. The *x*-axis represents the rich factor, and the y-axis represents the pathway name. DEGs, differentially expressed genes; KEGG, kyoto encyclopedia of genes and genomes.

To obtain a better insight into the interactions of functional genes by pathway-based analysis, all the genes were mapped to the KEGG database to classify the biological functions of the DEGs. Specific gene enrichment was observed in 79 pathways. Nineteen pathways showed DEG enrichment (Figure 5). It was observed that the pathway with the lowest *q* value and the largest number of DEGs was the pathway related to amino sugar and nucleotide sugar metabolism, followed by the pathways related to the synthesis of unsaturated fatty acids, nitrogen metabolism, and glycerolipid metabolism. Furthermore, there were 9 pathways with a *P*-value <0.05, such as “fatty acid degradation” (Supplementary Table S3). “Amino sugar and nucleotide sugar metabolism”, “Biosynthesis of unsaturated fatty acids”, and “Glycerolipid metabolism” showed an even greater significant enrichment (*P* < 0.01).

### Analysis of the Genes Related to Cordycepin Synthesis Pathway

**Figure 6:**
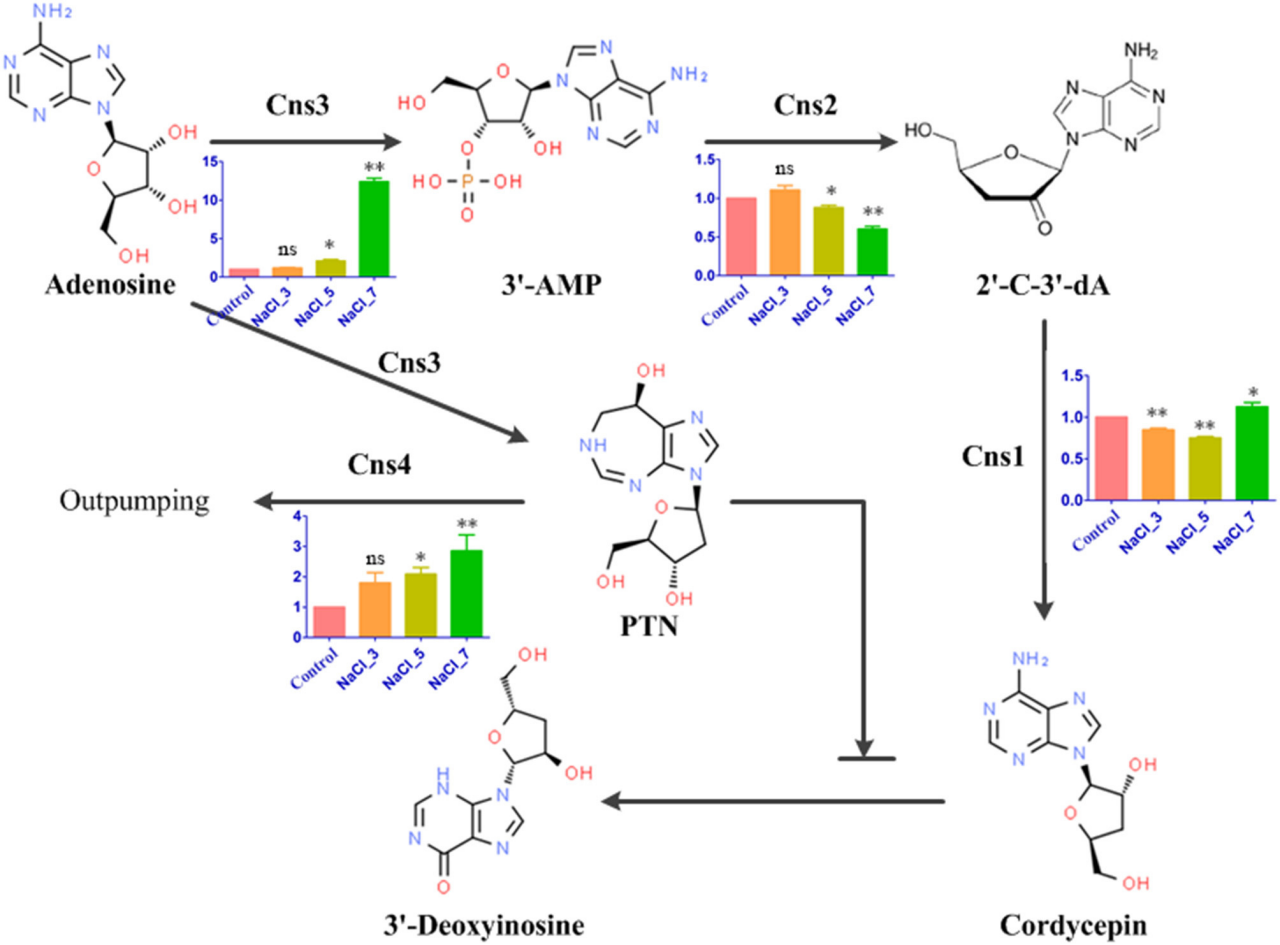
Cordycepin biosynthesis pathway in *C. militaris* and qRT-PCR results. Bar chart with 4 colors represents the relative expression levels of four genes in response to distinct salt treatments. The bars represent the average (±SE) of biological repeats. NaCl-3, 5, and 7 represent 3%, 5%, and 7% salt concentrations, respectively. Asterisks indicate statistically significant differences between groups (Student's *t*-test): * and ** indicating significance level was accepted at *P* ≤ 0.05 and *P* < 0.01, respectively, ns: no significant difference, as compared to control.

Since the cordycepin content was significantly improved by salt treatment, DEGs related to cordycepin synthesis were carefully identified by KEGG pathway analysis. According to the previously reported and generally accepted cordycepin biosynthesis pathway, this pathway started from adenosine to synthesize cordycepin and PTN (Figure 6). Four key genes involved in the cordycepin synthesis pathway, *Cns1, Cns2, Cns3*, and *Cns4*, were performed qRT-PCR validation. Though the fold changes of these genes in qRT-PCR were slightly different from that of RNA-Seq results, they shared a similar change tendency (Supplementary Figure S4). Based on the analysis of the cordycepin synthesis pathway and PCR results, the expression levels of *Cns1* and *Cns2* were decreased with the increase of salt concentration compared with the control, except *Cns1* on NaCl-7 group and *Cns2* on NaCl-3 group. *Cns1* and *Cns2* showed high expression levels on these two groups beyond that of the control. The dynamic changes of *Cns1* and *Cns2* expression levels illustrated that the response of these two genes to distinct salt treatments may differ. On the contrary, the expression level of *Cns3* was upregulated compared to the control, especially in the NaCl-7 group. This result indicated that the pathway of adenosine into 3′AMP might be greatly activated and finally increase the content of cordycepin under the treatment of 7% salt concentration. Additionally, the expression level of *Cns4* was also increased remarkably with the increment of salt concentration, revealing that salt treatment might likely facilitate the out pumping of PTN.

## Discussion

Considerable efforts have been put into the improvement of cordycepin production. The previous report showed that cordycepin content could be increased by nutrient stress (distinct carbon, nitrogen, phosphorous sources, and ratios), environmental stress (lighting time, temperature, pH, and shaking speed), and adding supplementation (inorganic salts, porcine liver extracts or vegetable oils, amino acids, or nucleoside analog) (8, 27–29). Though the pathway and related biosynthesis mechanisms of cordycepin have been well documented, the dependency of *C. militaris* activity on different salt concentrations in a medium remains unclear. To obtain more insight on *C. militaris* adaptations to salt stress conditions, precise physiological knowledge is needed. Therefore, a pre-experiment was carried out to evaluate the effect of salinity treatment on cordycepin production in *C. militaris*. In the present study, it was found that distinct salt concentration significantly inhibited the growth of *C. militaris* strains. The colony diameter and biomass of *C. militaris* strains were decreased with the increase of salt concentration (Figure 1 and Supplementary Figure S1). Obviously, *C. militaris* strain encountered osmotic stress due to the high-salt environment, and the salt tolerance of *C. militaris* cells might be essential for the long-term fermentation. High salinity represents a stress-induced high osmotic perturbation to cells since the excess salt disturbs osmotic potential and leads to metabolic toxicity (43). Analogously, clear growth defects under salinity conditions were also observed in *Streptomyces coelicolor*, and major changes occurred in the primary and secondary metabolism (Sugars, polyols, amino acids, nucleotides, and their derivatives) (33). The evolutions of varying morphological, physiological, and molecular mechanisms of adaptation are the main strategies of Halophilic fungi in reflection to osmotic stress conditions (44). Exactly, the morphological responses are meristematic growth, pigmentation, and changes in the cell wall and membrane composition (45). Moreover, the physiological responses consist of intracellular potassium and sodium ion content synthesis, accumulation of organic compatible solutes, and extracellular polysaccharide production. As for the molecular responses, it was reflected in the dynamic changes in gene expression related to physiological responses to enhanced salt concentration (44).

Contrary to the inhibited growth of *C. militaris* strain, the accumulation of cordycepin has a positive correlation with salt concentration after culturing in the PDA plates for 5 days (Figure 2). The yield of cordycepin on the NaCl-7 group was five-fold higher than that without salt supplementary. It was assumed that a high-salt environment might be optimal for cordycepin accumulation, whereas related molecular responses to an increased extracellular saline condition in *C. militaris* strain have not been identified. To figure out the metabolic pathway from the additive salt to cordycepin, four samples were collected, and transcriptome sequencing was performed based on RPKM metrics. Exposure of *C. militaris* cells to salt treatment results in a substantial transcriptional regulation, and 3,885 genes have altered expressions in three salinity treatment groups relative to the control (Figure 3). In the case of the NaCl-7 group, the number of DEGs reached a peak, revealing a greater degree of change in gene expressions in the high salt group than that in the low salt group. In fact, exposure of *C. militaris* cells to the high-salt environment implies threats of specific cation toxicity. So as to adapt to extracellular salinity, there is a transient induction of transcription that activates stress-protective genes (46, 47).

Furthermore, to analyze and classify the DEGs, the sequencing data were uploaded to the GO and KEGG databases (Figures 4, 5). GO classification results of DEGs showed that the salinity treatment may facilitate the metabolic process to produce more metabolites (cordycepin accumulation) and consequently activate membrane transportation (out-pumping of PTN). The cordycepin metabolism and the purine metabolism pathway have been elucidated in detail by comprehensive transcriptome and proteome analysis of *C. militaris* (21, 48). It was reported that cordycepin production can be increased by adding L-alanine, and a high transcriptional level of several genes encoding enzymes on the pathway of adenosine synthesis was also observed (8). Similarly, the majority of DEGs classified in “metabolic process” and “catalytic activity” items in this study showed different salt treatments may activate adenosine synthesis-related enzymes. And these enzymes catalyze series reactions related to adenosine synthesis and finally lead to the accumulation of cordycepin. Meanwhile, KEGG classification indicated that the most DEGs significantly enriched in the pathway of “Amino sugar and nucleotide sugar metabolism”, “Biosynthesis of unsaturated fatty acids”, and “Glycerolipid metabolism”. It was illustrated that salt treatment affected the specific energy metabolism and membrane composition changes. Most of the DEGs were relevant to the pathway of “Amino sugar and nucleotide sugar metabolism”, which revealed that the addition of salt might increase energy molecule production and further promote the cordycepin accumulation. It is generally accepted that cell membranes are one of the first targets to suffer from injurious effects of stress, leading to impaired cell membrane integrity and functions (49, 50). Plenty of studies have documented the modifications of cellular lipid profiles in response to osmotic shock (51, 52). In the present study, there were also a large number of DEGs enriched in the pathway of “Biosynthesis of unsaturated fatty acids” and “Glycerolipid metabolism”, which was consistent with the abovementioned Halophilic fungi in reflection to salt treatment condition by changing the membrane composition (morphological responses) (45). Additionally, it was reported that glycerolipid metabolism and high osmotic glycerol (HOG) signaling pathway enable micro-organisms to respond to various extracellular stimuli and also to adjust their cellular machinery to change the environments (53). Two isoenzymes of DL-glycerol-3-phosphatase in the glycerolipid metabolism pathway from *Saccharomyces cerevisiae* facilitate the ability of cells to expose to osmotic stress *via* the HOG pathway (54). Therefore, numbers of DEGs enriched in the pathway of glycerolipid metabolism in *C. militaris* strain indicated the addition of salt may achieve the same effect on that of *Saccharomyces cerevisiae*.

In the aforementioned context, cordycepin synthesis was mediated by a biosynthetic gene cluster, which consisted of gene *Cns1*-*Cns4* (21). Additionally, PTN, a purine analog with the activity of anticancer and adenosine deaminase inhibition, was noteworthily produced in coupling with cordycepin by *Cns3* (55). In the present study, dynamic changes of the transcription level of these four vital genes were observed based on the data obtained by transcriptome analysis. To further confirm the reliability and availability of these four DEGs, a real-time RT-PCR experiment was carried out. Overall, these qRT-PCR results showed that the DEGs transcription level was consistent with the result obtained by transcriptome analysis (Figure 6). Genes *Cns1, Cns3*, and *Cns4* were remarkably upregulated under high salt concentration which showed osmotic stress conditions may have beneficial to accumulating the cordycepin and transporting the PTN to the extracellular environment. *Cns2* was downregulated under distinct salt treatments (except on the Nacl-3 group), implying that the process of 3′AMP dephosphorylated to 2′-C-3′-dA by phosphohydrolase activity of *Cns2* was inhibited by salt treatment, and detailed molecular mechanism still needs further experimental validation. In addition, it has been reported that the yield of cordycepin was increased 2.7-fold by overexpression of the *Cns1*/*Cns2* fusion gene (21). Combined with the results in our study, it is worth trying that overexpression of the *Cns1*/*Cns2* or *Cns1*/*Cns2*/*Cns3* fusion gene *via* individually transformed the control strain of *C. militaris* under salt treatment. Furthermore, it has been reported that several genes can regulate osmoadaptation, such as salt-tolerant gene HOG and fatty acid metabolism gene delta-9 fatty acid desaturases (56, 57). Thus, it can be expected that the obtaining of industrial strains possesses salt tolerance and high cordycepin production *via* overexpression of the cordycepin biosynthetic gene and salt-tolerant gene. Interestingly, a significant upregulation of ribonucleotide reductase (RNR, 70.19-fold), 5′-Nucleotidase (*NT5E*, 63.43-fold), *purA* (Adenylosuccinate synthase, 8.43-fold), and Adenylate kinase (*ADEK*, 17.52-fold) gene expression was observed upon hypoxanthine treatment, which are downstream genes responsible for cordycepin biosynthesis in *Ophiocordyceps sinensis* (30). Analogous effects have also been achieved on several other potent growth supplements, such as amino acids, plant hormones, and vitamins. These results indicated both salt treatment and the addition of potent growth supplements can activate the cordycepin biosynthesis pathway leading to improved cordycepin content. Moreover, recent research first reported the transcriptome and proteomics of *Cordyceps kyushuensis* Kob, which is close relative to *C. militaris* (58). Similar to *C. militaris*, a single gene cluster containing *ck1-ck4*, which can synthesize both cordycepin and PTN, has been identified in *Cordyceps kyushuensis* Kob using BLAST. Therefore, it is worth exploiting the potential of *Cordyceps kyushuensis* Kob on the increment of cordycepin production in the near future apart from *C. militaris*. With the deep analysis of transcriptome and proteomics, more useful information to do contribution to the cosmetic and pharmaceutical industries can be provided by improving the yield of cordycepin.

## Conclusions

To better unravel the salt response mechanism of *C. militaris* and its effects on cordycepin content, our research performed a transcriptome analysis of *C. militaris* under the distinct salt treatments. The HPLC analysis depicted high salt treatment was beneficial to enhance cordycepin production. Combined with GO analysis and KEGG pathway enrichment, the upregulated transcriptome level of genes responsible for the biosynthetic pathways of energy generation and lipid metabolism might be the major reason for the accumulation of cordycepin. Finally, mRNA expression analysis of four genes involved in the cordycepin biosynthesis pathway was carried out by RT-PCR, and the results showed that the transcription level of these genes was consistent with that of transcriptome analysis. Taken together, our study provides a global transcriptome characterization of the osmotic stress adaptation process in *C. militaris* and paves the way for constructing industrial strains that possess salt tolerance and high cordycepin production.

## Data Availability Statement

The datasets presented in this study can be found in online repositories. The names of the repository/repositories and accession number(s) can be found below: https://www.ncbi.nlm.nih.gov/, PRJNA770191.

## Author Contributions

GL: conceptualization, visualization, and writing-original draft preparation. YZ: conceptualization. XC and YC: project administration. BZ and XL: supervision and funding acquisition. BH: writing review, editing, and funding acquisition. All authors contributed to the article and approved the submitted version.

## Funding

This research was funded by the Natural Science Foundation of Jiangxi Province (Grant Nos. 20202BABL203043, 20202BAB215003, and 20212BAB215005) and Jiangxi Double Thousand Plan Cultivation Program for Distinguished Talents in Scientific and Technological Innovation (jxsq2019201011).

## Conflict of Interest

The authors declare that the research was conducted in the absence of any commercial or financial relationships that could be construed as a potential conflict of interest.

## Publisher's Note

All claims expressed in this article are solely those of the authors and do not necessarily represent those of their affiliated organizations, or those of the publisher, the editors and the reviewers. Any product that may be evaluated in this article, or claim that may be made by its manufacturer, is not guaranteed or endorsed by the publisher.

## Supplementary Material

Figure S1 Determination of the biomass of the *C. militaris* mycelia under salt treatment. 0, 3, 5, and 7% are equivalent to the control, slight, moderate, and severe salt treatment, respectively.

Figure S2 HPLC comparison of cordycepin production among the control and three salt treatment groups. 0, 3, 5, and 7% represent the control, slight, moderate, and severe salt-treated groups, respectively.

Figure S3 Heatmap of the hierarchical clustering of the DEGs. A, B, and C represent Control vs. NaCl-3, -5, and -7, respectively, (the *x*-axis represents each compared sample; the *y*-axis represents the DEGs. The coloring indicates the fold change: high, green; low, red). DEGs, differentially expressed genes.

Figure S4 A comparison of transcriptional level change of genes (*Cns1-Cns4*) between transcriptome analysis and RT-PCR validation.

Table S1 qRT-PCR primers used in this study.

Table S2 The identification of significant DEGs among four treatments (Control vs. NaCl-3, -5, and -7). DEGs, differentially expressed genes.

Table S3 KEGG pathway enrichment of DEGs. DEGs, differentially expressed genes; KEGG, kyoto encyclopedia of genes and genomes.
