# Supplementary material for: Transcriptomic Responses of Cordyceps militaris to Salt Treatment During Cordycepins Production
Source: Front Nutr. 2021 Dec 23;8:793795. doi: 10.3389/fnut.2021.793795 (PMC8733472; doi:10.3389/fnut.2021.793795)
Supplement: Figure S1 — Determination of the biomass of the C. militaris mycelia under salt treatment. 0, 3, 5, and 7% are equivalent to the control, slight, moderate, and severe salt treatment, respectively. [file Data_Sheet_1.ZIP › Table S1.docx]

**Supplementary Table S1. qRT-PCR Primers used in this study**

| **Primer** | **Sequence (5′-3′)** | **Purpose** |
| --- | --- | --- |
| Cns1-F | CGAGATCATTGCCGAGATGAA | qRT-PCR of Cns1 |
| Cns1-R | GTGTAGTCGTGGTTGGTCTTAC | qRT-PCR of Cns1 |
| Cns2-F | GGCAGTCAAGTAGACATGGAG | qRT-PCR of Cns2 |
| Cns2-R | ACCGTTTATCCGAGGAGAGTA | qRT-PCR of Cns2 |
| Cns3-F | CCCTCTATGATCAGCACAAGTC | qRT-PCR of Cns3 |
| Cns3-R | TAGGCACCATTGCCCTTTAG | qRT-PCR of Cns3 |
| Cns4-F | ACACAGTGGTACTGTTGATGAG | qRT-PCR of Cns4 |
| Cns4-R  GAPDH-F  GAPDH-R | CGATGAGTGTGAGAAGCCATAG  GACAACATCCAGGGTATCACTAAGC  GGTCTCCTCGTAGATCATGGCA | qRT-PCR of Cns4  qRT-PCR of the reference gene  qRT-PCR of the reference gene |
