# Supplementary figures and images for: Transcriptomic Responses of Cordyceps militaris to Salt Treatment During Cordycepins Production
Source: Front Nutr. 2021 Dec 23;8:793795. doi: 10.3389/fnut.2021.793795 (PMC8733472; doi:10.3389/fnut.2021.793795)

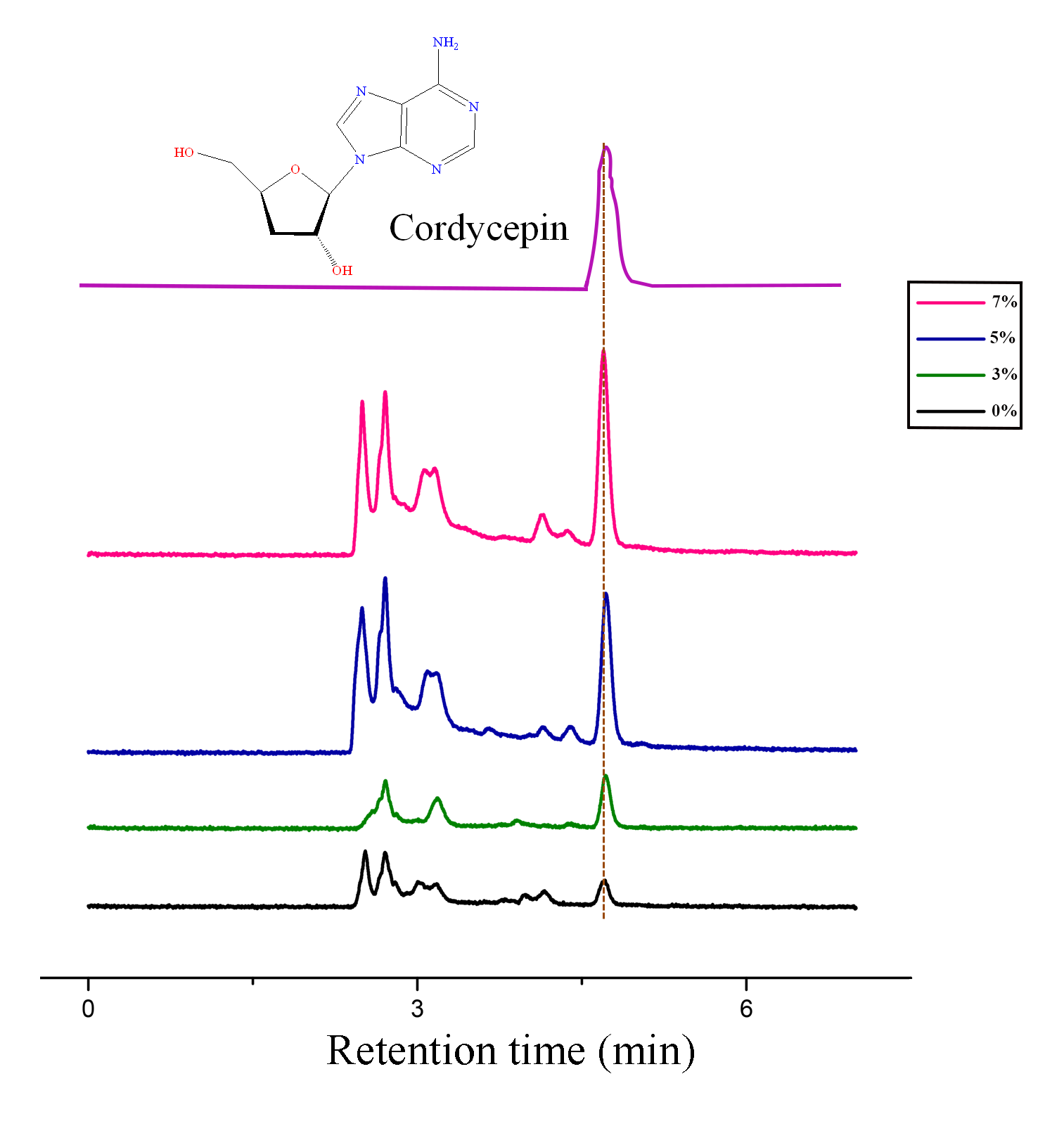

Supplement: Figure S1 — Determination of the biomass of the C. militaris mycelia under salt treatment. 0, 3, 5, and 7% are equivalent to the control, slight, moderate, and severe salt treatment, respectively. [file Data_Sheet_1.ZIP › Figure S2.tif]

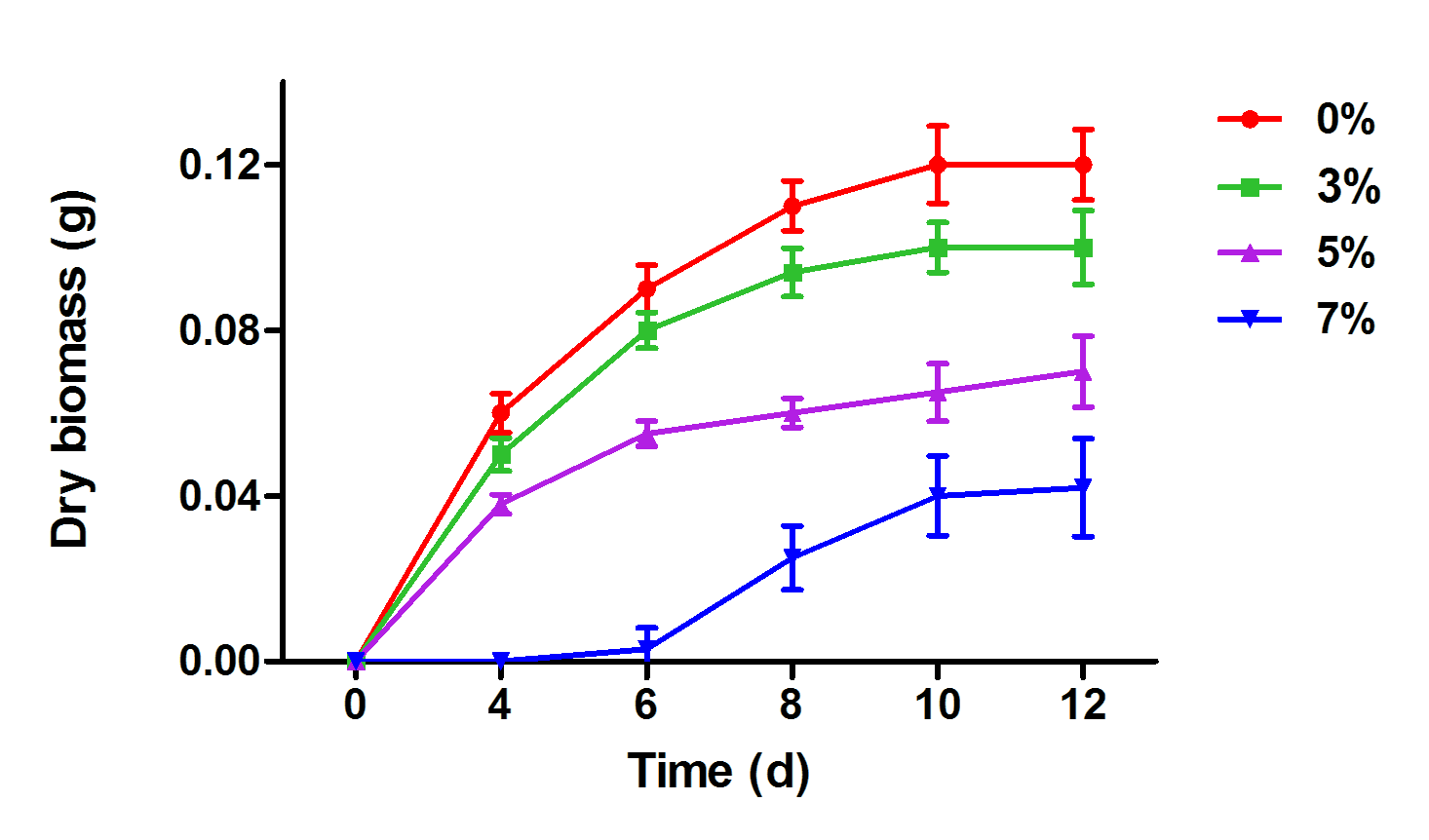

Supplement: Figure S1 — Determination of the biomass of the C. militaris mycelia under salt treatment. 0, 3, 5, and 7% are equivalent to the control, slight, moderate, and severe salt treatment, respectively. [file Data_Sheet_1.ZIP › Figure S1.tif]

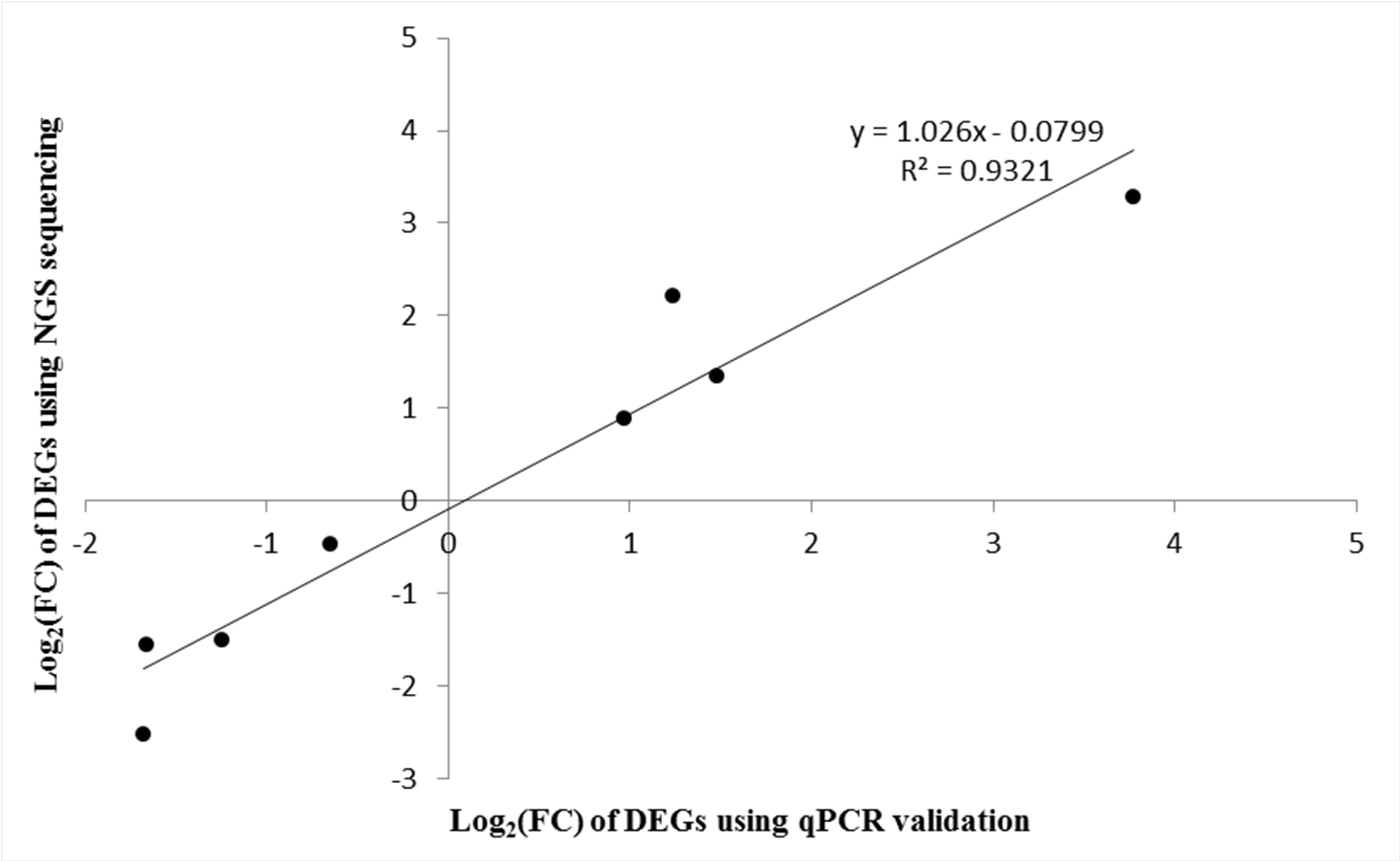

Supplement: Figure S1 — Determination of the biomass of the C. militaris mycelia under salt treatment. 0, 3, 5, and 7% are equivalent to the control, slight, moderate, and severe salt treatment, respectively. [file Data_Sheet_1.ZIP › Figure S4.tif]

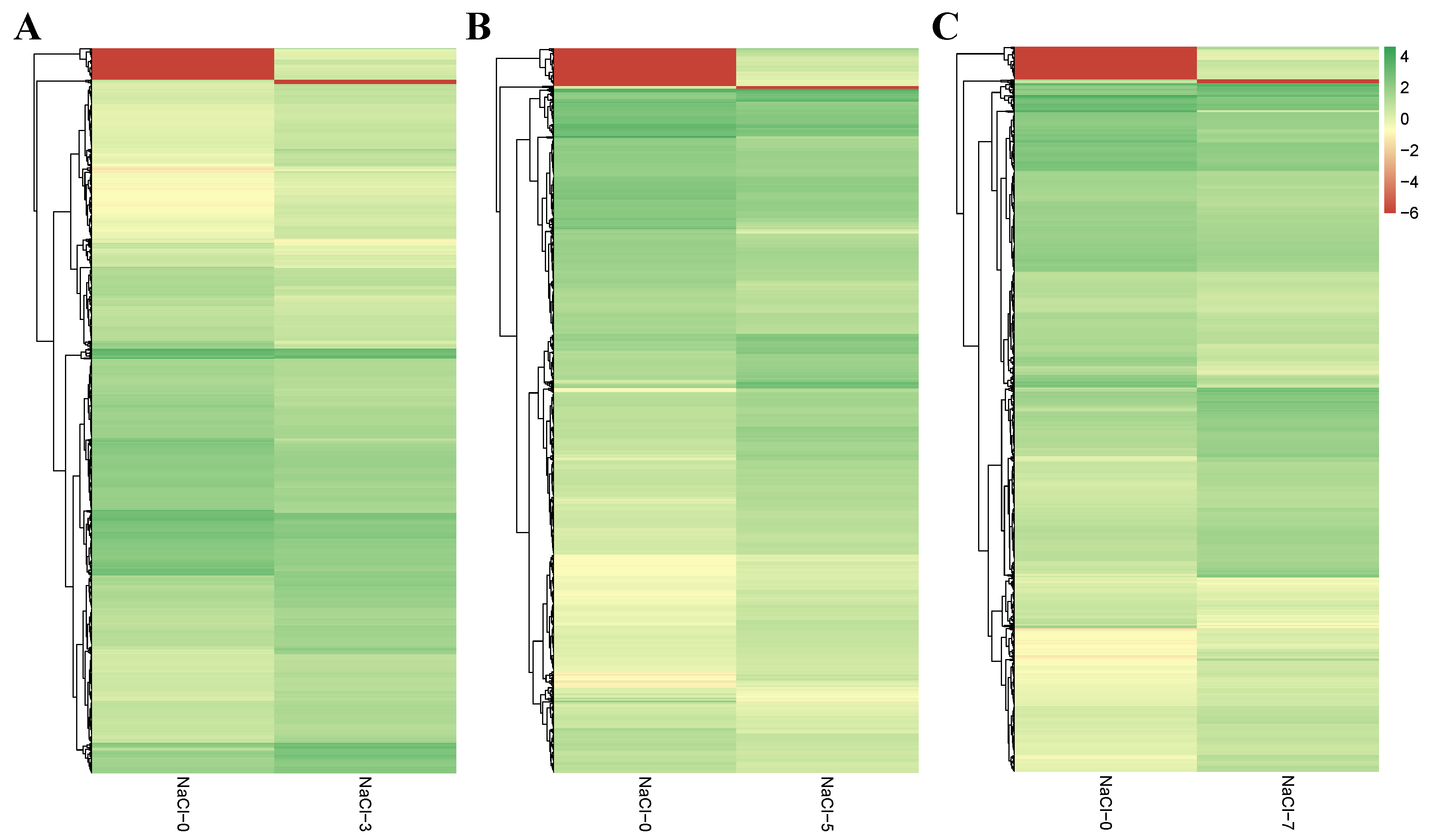

Supplement: Figure S1 — Determination of the biomass of the C. militaris mycelia under salt treatment. 0, 3, 5, and 7% are equivalent to the control, slight, moderate, and severe salt treatment, respectively. [file Data_Sheet_1.ZIP › Figure S3.tif]
